# Lobectomy Can Improve the Survival of Patients With Non-small Cell Lung Cancer With Lung Oligometastatic

**DOI:** 10.3389/fsurg.2021.685186

**Published:** 2021-07-05

**Authors:** Lingwei Wang, Fanglei Jiao, Lin Dong, Qinchuan Li, Gang Liu, Xuefei Hu

**Affiliations:** ^1^Tongji University School of Medicine, Tongji University, Shanghai, China; ^2^Department of Thoracic Surgery, East Hospital of Tongji University School of Medicine, Shanghai, China; ^3^Thoracic Department of Shanghai Pulmonary Hospital, Tongji University School of Medicine, Shanghai, China

**Keywords:** NSCLC, single-organ metastasis, multiple metastases, lobectomy, SEER

## Abstract

**Background:** This study was to evaluate the value of lobectomy in the prognosis of Non-small cell lung cancer (NSCLC) patients with primary metastasis based on the Surveillance Epidemiology and End Results (SEER) database.

**Methods:** This was a population-based retrospective study and the clinical data were collected from the National Cancer Institute's SEER database between 2010 and 2015. The effects of pulmonary surgery and surgical procedures on lung cancer-specific survival (LCSS) and overall survival (OS) were assessed, and the COX regression models were employed to evaluate the survival of primary surgery in patients with primary metastatic NSCLC (pmNSCLC) and the survival of surgical procedure in pmNSCLC patients.

**Results:** A total of 55,717 patients diagnosed with pmNSCLC between 2010 and 2015 were enrolled, and pulmonary surgery was indicated in 1,575 (2.83%) patients. Surgery was an independent risk factor for LCSS (*P* < 0.001, HR 0.658, 95%CI: 0.637–0.680) and OS (*P* < 0.001, HR 0.665, 95%CI: 0.644–0.686) of pmNSCLC patients. The surgery was associated with better OS (*P* < 0.001, HR 0.678, 95%CI: 0.657–0.699). The site of metastasis was also related to the survival after primary tumor surgery (*P* = 0.001). As compared to the sublobectomy and pneumonectomy, lobectomy improved the LCSS for NSCLC patients with single-organ metastasis, rather than multiple metastases (*P* < 0.001). In patients receiving sublobectomy, lobectomy, and pneumonectomy, the median LCSS was 12, 28, and 13 months, respectively, and the 5-year LCSS rate was 14.39, 32.06, and 17.24%, respectively.

**Conclusion:** The effect of locoregional surgery on the survival of pmNSCLC patients with single-organ metastasis has been underestimated, and lobectomy may be a preferred treatment for patients with single-lung metastasis.

## Introduction

Lung cancer is the most common cancer and the leading cause of cancer-related deaths worldwide (1, 2). In clinical practice, most primary lung cancers (about 80%) are non-small cell lung cancer (NSCLC). Among initially diagnosed patients, ~50% of NSCLC patients are diagnosed with stage IV disease and have distant metastasis (3). The brain is the most common site of lung cancer metastasis (4), followed by the bone, lung, adrenal gland, and liver (5).

Radical resection is still the most effective treatment for early-stage lung cancer. Systemic therapies are recommended for patients with stage IV lung cancer including chemotherapy, radiotherapy, targeted therapy, and immunotherapy. The efficacy of surgical management for advanced lung cancer remains controversial. However, surgery is generally not a treatment of choice for primary metastatic non-small cell lung cancer (pmNSCLC). Studies have reported that surgical removal of primary lesions can improve the prognosis of ovarian and gastric carcinoma (6, 7), which has facilitated the investigation of the efficacy of surgery in improving the overall survival (OS) of pmNSCLC patients.

Several studies have revealed that the resection of primary lung tumor may be an option for NSCLC patients with single-organ metastasis (no more than five metastases in a single organ or oligometastatic) undergoing effective local therapy for distant metastasis (8–12). Wang et al. found there was no significant difference in the survival rate among patients treated with different surgical procedures (13). The expansion of these results is limited by the study design, small sample size, and heterogeneity of the study population.

Hence, the present study was to evaluate the efficacy of lobectomy for NSCLC with single-organ metastasis in the population-based Surveillance, Epidemiology, and End Results (SEER) database, which includes the information regarding the primary treatment, metastatic organ, chemotherapy, radiotherapy, T stage, N stage, and tumor size. The OS and LCSS were assessed based on the patients' characteristics.

## Patients and Methods

### Study Population

Data of lung cancer patients with distant metastasis diagnosed between 2010 and 2015 were extracted using SEER^*^Stat software version 8.3.5. Patients were diagnosed according to the International Classification of Disease histology code for Oncology (ICD-O-3) with adenocarcinoma (8,140–8,147, 8,255, 8,260, 8,310, 8,323, 8,480, 8,481, 8,490, 8,550, 8,572), squamous cell carcinoma (8,050–8,052, 8,070–8,078), large-cell carcinoma (8,012–8,014), and other pathologies including undifferentiated tumors (8,020–8,022) and carcinomas not otherwise specified (8,010). The exclusion criteria were as follows: (1) patients had more than one primary tumors; (2) patients had no information on the survival; (3) patients had incomplete clinicopathological information; (4) the diagnosis was not pathologically confirmed; (5) patients had no information regarding the indication for primary tumor surgery.

Demographic and clinical variables included age at diagnosis, sex, histology, AJCC T stage, tumor grade, AJCC N stage, and surgical procedure. Age at diagnosis was stratified by ≤ 65 and >65 years. The TNM stage was reclassified for each patient based on the primary tumor size and extent of invasion according to the TNM classification for lung cancer (8th edition) using R version 3.4.3 software. The surgical procedures for the primary lesion included pneumonectomy, lobectomy, and sublobectomy.

### Statistical Analysis

The demographic, pathologic, and surgical characteristics were compared between patients with and without surgery using the Pearson χ2 test. The OS and LCSS were analyzed with the Kaplan-Meier method and the survival with the log-rank test. Cox proportional hazards model was employed for univariate and multivariate analyses. Variables that were significantly associated with the survival in univariate analysis were included in the multivariate Cox analysis. A value of two-sided *P* < 0.05 was considered statistically significant. Statistical analysis was performed with SPSS version 25.0 (SPSS, Chicago, IL). GraphPad Prism 8.0 (GraphPad Software, San Diego, CA) was used to plot the survival curve.

## Results

### Clinical Characteristics of Patients

A total of 55,717 patients diagnosed with pmNSCLC in the United States between 2010 and 2015 were enrolled in the present study. Among them, 1,575 (2.83%) received surgical treatment, while 54,142 (97.17%) received conservative treatment. The clinical characteristics of these patients are summarized in Table 1. The tumor in patients receiving surgery was more likely to be Grade III or IV tumor, and less likely to be treated by radiotherapy as compared to the non-surgery group (40.03 vs. 49.11%, 2.92 vs. 1.36%). More than half of patients received systemic chemotherapy in both groups, however, the patients in the surgery group were more likely to receive chemotherapy than in the non-surgery group (57.65 vs. 53.01%, *P* < 0.001). Based on the AJCC staging, the earlier the staging, the higher the proportion of patients receiving surgery was in respect of T stage and N stage. Among patients with organ metastasis at the first diagnosis of NSCLC, patients with brain or lung metastasis were more likely to receive locoregional surgery than those with bone or liver metastasis or multiple metastases (multiple organ metastases or more than five metastases in a single organ).

**Table 1:** Demographics of all patients with primary metastatic lung cancer.

		**Surgery (%)**		
**Variables**	**All**	**No**	**Yes**	***P***
Age (years)				<0.001
≤ 65	25,116	24,232 (44.76)	884 (56.13)	
>65	30,601	29,910 (55.24)	691 (43.87)	
Gender				<0.001
Male	30,775	29,974 (55.36)	801 (50.86)	
Female	24,942	24,168 (44.64)	774 (49.14)	
Histology				<0.001
Adenocarcinoma	32,684	31,742 (58.63)	942 (59.81)	
Squamous	10,248	9,989 (18.45)	259 (16.44)	
Others	12,785	12,411 (22.92)	374 (23.75)	
Grade				<0.001
I	1,308	1,169 (2.16)	139 (8.83)	
II	6,044	5,617 (10.37)	427 (27.11)	
III	14,841	14,201 (26.23)	640 (40.63)	
IV	785	739 (1.36)	46 (2.92)	
Unknown	32,739	32,416 (59.88)	323 (20.51)	
Radiation				0.017
No	28,402	27,552 (50.89)	850 (53.97)	
Yes	27,315	26,590 (49.11)	725 (40.03)	
Chemotherapy				<0.001
No	26,106	25,439 (46.99)	667 (42.35)	
Yes	29,611	28,703 (53.01)	908 (57.65)	
T stage				0.001
T1	6,602	6,376 (11.78)	226 (14.35)	
T2	10,148	9,840 (18.17)	308 (19.56)	
T3	14,199	13,791 (25.47)	408 (25.90)	
T4	24,768	24,135 (44.58)	633 (40.19)	
N stage				<0.001
N0	12,420	11,721 (21.65)	699 (44.38)	
N1	4,478	4,280 (7.91)	198 (12.57)	
N2	24,891	24,396 (45.06)	495 (31.43)	
N3	11,428	11,294 (20.86)	134 (8.51)	
Nx	2,500	2,451 (4.52)	49 (3.11)	
Metastasis site				<0.001
Bone	11,863	11,622 (21.47)	241 (15.30)	
Brain	8,886	8,436 (15.58)	450 (28.57)	
Liver	3,467	3,387 (6.26)	80 (5.08)	
Lung	10,717	10,177 (18.79)	540 (34.29)	
Multiple-site	20,784	20,520 (37.90)	264 (16.76)	

### Univariate and Multivariate Analysis of All Patients

**Figure 1:**
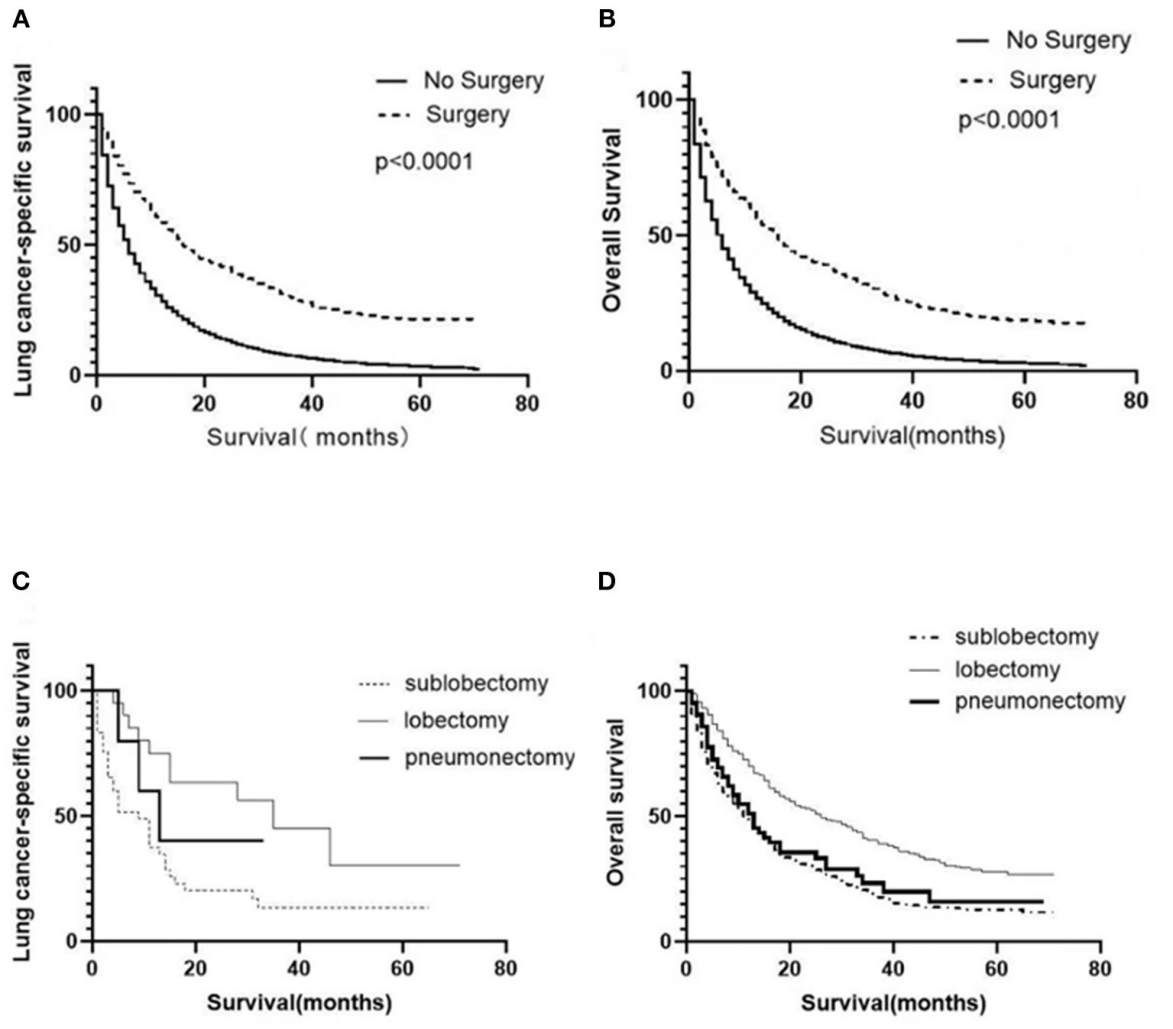
Survival of NSCLC patients with metastasis. Survival curves showed the LCSS **(A)** and OS **(B)** of all patients in the surgery and non-surgery groups. Survival curves showed the LCSS **(C)** and OS **(D)** of surgical patients receiving different surgical procedures.

Univariate analysis was employed to identify the potential factors affecting LCSS and OS of patients with organ metastasis. As shown in Tables 2, 3, although the cancer cell differentiation had no significant influence on the prognosis (LCSS: *P* = 0.277, OS: *P* = 0.251), the poor differentiation predicted a worse prognosis than the well-differentiation. The risk of cancer-related death increased with age (*P* < 0.001), while the male patients were at a higher risk as compared to female patients (*P* < 0.001). Similarly, the younger and female patients with metastasis achieved better OS (*P* < 0.001). In respect of pathology, adenocarcinoma seemed to be associated with better LCSS and OS. Both local radiotherapy and systemic chemotherapy prolonged the LCSS and OS of the pmNSCLC patients. Tumors with higher T stage and N stage had poorer LCSS and OS. The NSCLC patients with liver or multiple metastasis had worse LCSS and OS as compared to those with bone, brain, or lung involvement. The LCSS curves of patients in the surgery and non-surgery groups are shown in Figure 1A. Primary surgery significantly improved the LCSS of patients with primary metastasis (*P* < 0.001, HR 0.449, 95%CI: 0.421–0.479). The median LCSS was 6 months in the non-surgery group and 16 months in the surgery group (*P* < 0.01). In the non-surgery group and surgery group, the 3-year LCSS was 7.44 and 29.92%, respectively, and the 5-year LCSS was 3.41 and 21.59%, respectively. The median OS was 6 months in the non-surgery group and 16 months in the surgery group(*P* < 0.01). In the non-surgery group and surgery group, the 3-year OS was 6.58 and 27.57%, respectively, and the 5-year OS was 2.89 and 18.87%, respectively. The OS curves of patients in both groups are shown in Figure 1B. The surgery was associated with better OS (*P* < 0.001, HR 0.678, 95%CI: 0.657–0.699).

**Table 2:** Univariate and multivariate analysis between clinicopathological characteristics for LCSS of all patients.

	**Univariate analysis**		**Multivariate analysis**	
**Variables**	**HR (95% CI)**	***P*-value**	**HR (95% CI)**	***P*-value**
Age	1.274 (1.251–1.299)	<0.001	1.088 (1.067–1.109)	<0.001
Gender	0.813 (0.798–0.828)	<0.001	0.819 (0.803–0.835)	<0.001
Histology	1.159 (1.146–1.172)	<0.001	1.102 (1.090–1.114)	<0.001
Grade	1.004 (0.997–1.011)	0.277	–	–
Radiation	0.856 (0.840–0.872)	<0.001	0.977 (0.958–0.996)	0.016
Chemotherapy	0.384 (0.377–0.391)	<0.001	0.381 (0.373–0.388)	<0.001
T stage	1.073 (1.064–1.083)	<0.001	1.055 (1.045–1.065)	<0.001
N stage	1.075 (1.066–1.083)	<0.001	1.101 (1.092–1.110)	<0.001
Metastasis site	1.047 (1.040–1.053)	<0.001	1.036 (1.030–1.043)	<0.001
Primary surgery	0.449 (0.421–0.479)	<0.001	0.658 (0.637–0.680)	<0.001

**Table 3:** Univariate and multivariate analysis of clinicopathological characteristics for OS of all patients.

	**Univariate analysis**		**Multivariate analysis**	
**Variables**	**HR (95% CI)**	***P*-value**	**HR (95% CI)**	***P*-value**
Age	1.296 (1.273–1.320)	<0.001	1.101 (1.080–1.122)	<0.001
Gender	0.810 (0.795–0.825)	<0.001	0.813 (0.798–0.829)	<0.001
Histology	1.159 (1.147–1.172)	<0.001	1.101 (1.089–1.113)	<0.001
Grade	1.004 (0.997–1.011)	0.251	–	–
Radiation	0.837 (0.822–0.852)	<0.001	0.958 (0.940–0.976)	<0.001
Chemotherapy	0.378 (0.371–0.385)	<0.001	0.377 (0.370–0.384)	<0.001
T stage	1.072 (1.062–1.081)	<0.001	1.053 (1.044–1.063)	<0.001
N stage	1.071 (1.063–1.080)	<0.001	1.099 (1.090–1.108)	<0.001
Metastasis site	1.046 (1.040–1.052)	<0.001	1.035 (1.029–1.042)	<0.001
Primary surgery	0.678 (0.657–0.699)	<0.001	0.665 (0.644–0.686)	<0.001

Multivariate analysis was employed to identify the potential factors affecting LCSS and OS in patients with primary metastasis. As shown in Tables 2, 3, advanced age (>65 years), male gender, higher T stage, higher N stage, no radiotherapy, no chemotherapy, liver or multiple metastasis, and no surgery were independent risk factors for both LCSS and OS (*P* < 0.005).

### Univariate and Multivariate Analysis of Surgically Treated Patients

**Figure 2:**
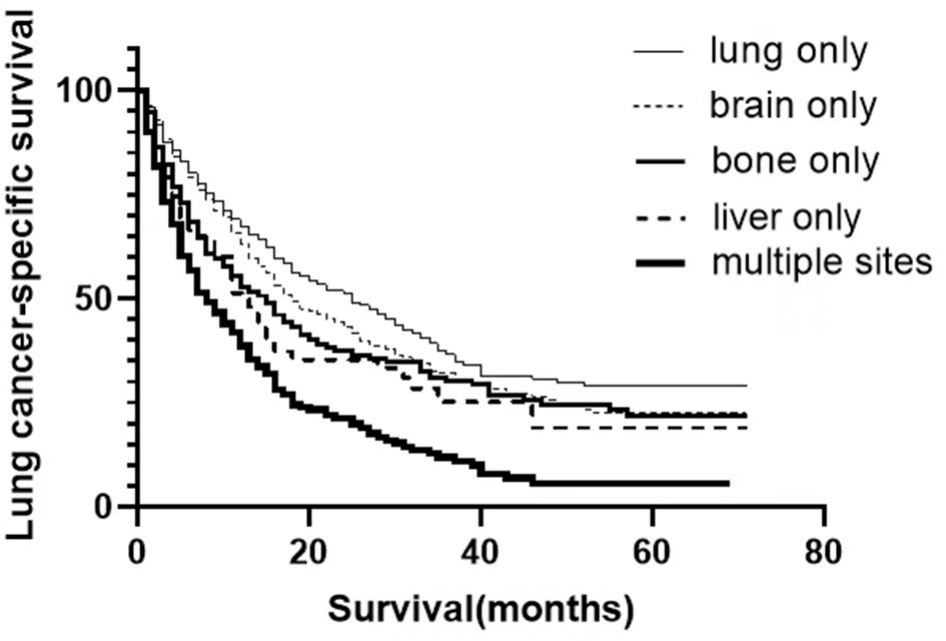
LCSS curves of NSCLC patients with different site metastasis after primary surgery.

**Figure 3:**
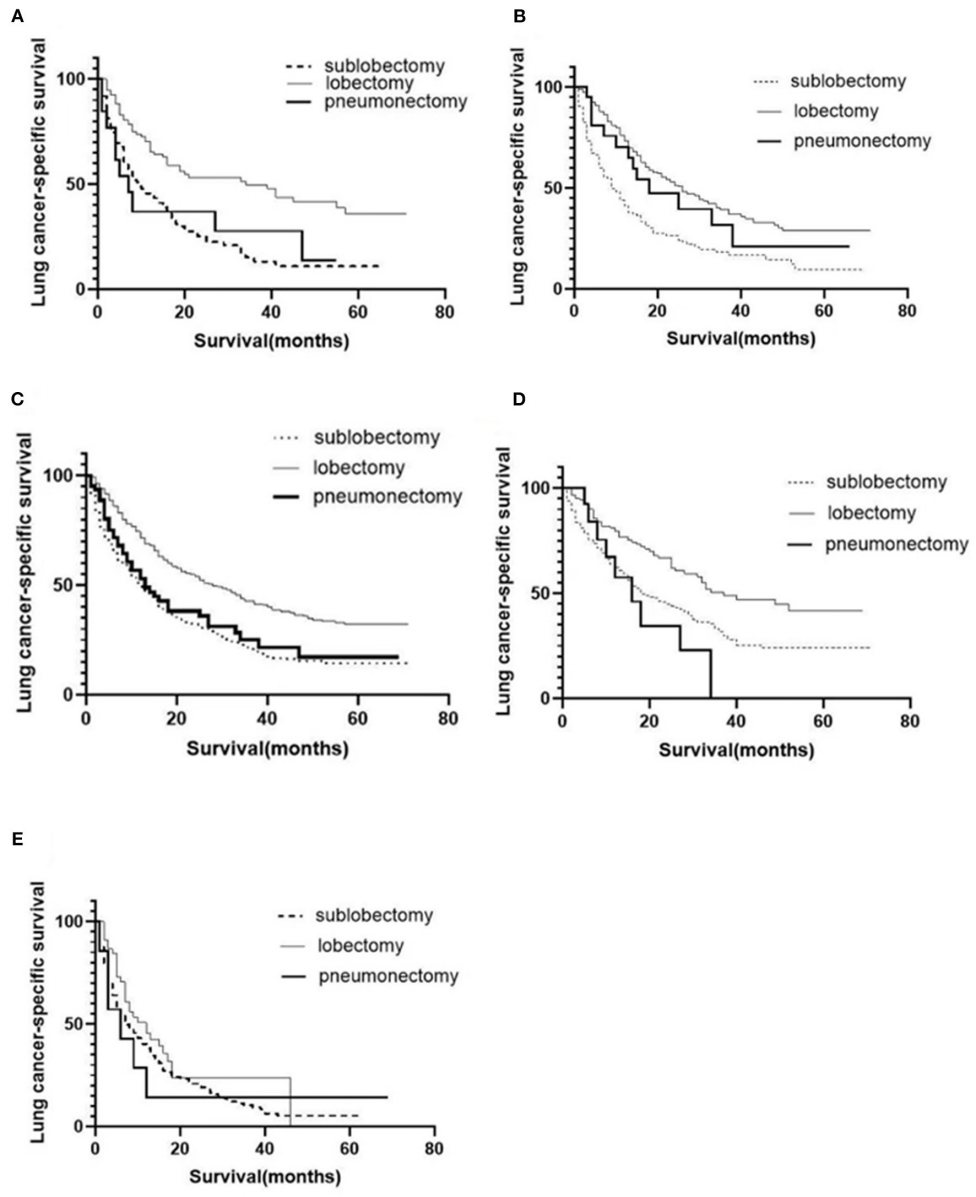
Postoperative LCSS curves of patients receiving different surgical procedures after classification by organs involved. LCSS curves of bone metastasis **(A)**; brain metastasis **(B)**; liver metastasis **(C)**; lung metastasis **(D)**; multiple metastases **(E)**.

A total of 1,575 patients with primary metastasis received surgical treatment. Among them, 906 patients underwent sublobectomy, 605 patients underwent lobectomy, and 64 patients underwent pneumonectomy. The univariate analysis was used to analyze the clinical characteristics affecting the LCSS and OS in surgically treated patients with distant organ metastasis. As shown in Tables 4, 5, histology and radiotherapy had no significant effects on the LCSS and OS (*P* > 0.05). In this cohort, older and male patients had worse survival (LCSS and OS). The grade had no relationship with the survival of all the patients, but a significant association was noted in the surgically treated patients (*P* = 0.015). Chemotherapy, as a systemic treatment, achieved better survival in surgically treated patients. In the surgery group, tumors of higher T stage and N stage also had poorer LCSS and OS. The site of metastasis was also related to the survival after primary tumor surgery (*P* = 0.001). The LCSS curves of metastasis sites are shown in Figure 2. Patients with lung metastasis had the best survival, while patients with multiple metastasis had the worst survival. The median LCSS was 25 months in patients with lung metastasis, 18 months in patients with brain metastasis, 15 months in patients with bone metastasis, 13 months in patients with liver metastasis, and 8 months in patients with multiple metastasis. The surgical procedure of primary lesion also significantly affected the LCSS (*P* < 0.001, HR 0.592,95%CI: 0.524–0.668) and OS (*P* < 0.001, HR 0.602, 95%CI: 0.535–0.676). The median LCSS was 12 months in patients receiving sublobectomy, 28 months in those receiving lobectomy, and 13 months in those receiving pneumonectomy. As compared to the sublobectomy and pneumonectomy, lobectomy significantly improved the LCSS and OS of patients. The survival curves are shown in Figures 1C,D. There were no significant differences between the sublobectomy group and the pneumonectomy group in the LCSS (*P* = 0.350) and OS (*P* = 0.369). In the sublobectomy, lobectomy, and pneumonectomy groups, the 3-year LCSS was 20.85, 42.99, and 25.14%, respectively, and the 5-year LCSS was 14.39, 32.06, and 17.24%, respectively. The median OS was 11 months in the sublobectomy group, 26 months in the lobectomy group, and 13 months in the pneumonectomy group. The 3-year OS was 18.84% in the sublobectomy group, was 40.38% in the lobectomy group, and 23.31% in the pneumonectomy group. In the sublobectomy, lobectomy, and pneumonectomy groups, the 5-year OS was 12.74, 28.01, and 15.99%, respectively. The LCSS curves of patients receiving different surgical procedures are shown in Figure 3. As compared to the sublobectomy and pneumonectomy groups, lobectomy improved the LCSS for NSCLC patients with single-organ metastasis, rather than multiple metastases. The median LCSS in the surgically treated patients with metastasis to the bone, brain, liver, lung, and multiple organs was 15, 18, 13, 25, and 8 months, respectively.

**Table 4:** Univariate and multivariate analysis of clinicopathological characteristics for LCSS of the surgical patients.

	**Univariate analysis**		**Multivariate analysis**	
**Variables**	**HR (95% CI)**	***P*-value**	**HR (95% CI)**	***P*-value**
Age	1.330 (1.171–1.510)	<0.001	1.176 (1.032–1.341)	0.015
Gender	0.653 (0.574–0.742)	<0.001	0.652 (0.571–0.744)	<0.001
Histology	1.027 (0.961–1.098)	0.426	–	–
Grade	1.072 (1.013–1.134)	0.015	1.112 (1.049–1.179)	<0.001
Radiation	1.014 (0.893–1.151)	0.836	–	–
Chemotherapy	0.650 (0.572–0.738)	<0.001	0.568 (0.496–0.650)	<0.001
T stage	1.150 (1.082–1.222)	<0.001	1.124 (1.055–1.198)	<0.001
N stage	1.273 (1.207–1.341)	<0.001	1.242 (1.178–1.309)	<0.001
Metastasis site	1.086 (1.035–1.139)	0.001	1.001 (0.950–1.055)	0.973
Surgery approach	0.592 (0.524–0.668)	<0.001	0.606 (0.533–0.689)	<0.001

**Table 5:** Univariate and multivariate analysis of clinicopathological characteristics for OS of the surgical patients.

	**Univariate analysis**		**Multivariate analysis**	
**Variables**	**HR (95% CI)**	***P*-value**	**HR (95% CI)**	***P*-value**
Age	1.363 (1.206–1.542)	<0.001	1.206 (1.063–1.369)	0.004
Gender	0.647 (0.572–0.733)	<0.001	0.643 (0.566–0.730)	<0.001
Histology	1.025 (0.961–1.092)	0.458	–	–
Grade	1.077 (1.020–1.137)	0.008	1.116 (1.055–1.181)	<0.001
Radiation	1.014 (0.897–1.146)	0.829	–	–
Chemotherapy	0.628 (0.555–0.710)	<0.001	0.552 (0.484–0.629)	<0.001
T stage	1.140 (1.075–1.208)	<0.001	1.120 (1.053–1.191)	<0.001
N stage	1.252 (1.190–1.317)	<0.001	1.225 (1.164–1.289)	<0.001
Metastasis site	1.083 (1.034–1.134)	0.001	1.001 (0.952–1.054)	0.959
Surgery approach	0.602 (0.535–0.676)	<0.001	0.612 (0.540–0.693)	<0.001

## Discussion

In recent years, with the development of genetic technology and targeted treatment, molecular targeted therapy plays an important role in the management of stage IV NSCLC (14). In our study, the information about gene mutations in lung cancer, such as gene mutation of the epidermal growth factor receptor tyrosine kinase domain which is routinely examined in the malignancies from the SEER database was unclear. Thus, the response of these patients to targeted therapy was unclear. In addition, radiofrequency ablation and stereotactic radiotherapy are increasingly introduced to treat advanced lung cancer (15, 16). These individualized treatments are less invasive and may improve the quality of life and reduce adverse events (17). In the present analysis, radiotherapy improved the survival of all patients with pmNSCLC but did not influence the survival of surgically treated patients. That is, the pmNSCLC patients will not benefit from the radiotherapy. Thus, it remains controversial whether primary surgery is necessary for stage IV NSCLC.

In clinical practice, cancers with distant metastasis are considered incurable, and thus usually systemic chemotherapy is administered for palliative treatment. Some studies have reported that locoregional surgery may improve the survival of patients with relatively limited metastasis (8, 10, 11). The NSCLC staging system (eighth edition) emphasizes the advantages of local therapies (including surgical resection and radiotherapy) in the treatment of metastatic lung cancer (18). Endo et al. reported that, in NSCLC patients with limited distant metastasis, the 5-year OS rate was 40% after primary lung lesion resection (11). Luketich et al. investigated the prognosis of NSCLC patients with adrenal metastasis who received chemotherapy alone or surgical resection after chemotherapy. Their results showed the median survival time (MST) was 8.5 months in the chemotherapy group, and 31 months in the combination treatment group, showing a significant difference (19). In univariate and multivariate analyses, our results showed surgical resection significantly improved the prognosis of NSCLC patients with distant single-organ metastasis. The MST was 6 months in the non-surgery group and 16 months in the surgery group. The 5-year OS rate was 2.89% in the non-surgery group and 18.87% in the surgery group. The site of metastasis also affected the survival after primary tumor surgery. Patients with lung metastasis alone had the best survival, while those with multiple metastasis had the worst survival. The median LCSS was 25 months in patients with lung metastasis, 18 months in those with brain metastasis, 15 months in those with bone metastasis, 13 months in those with liver metastasis, and 8 months in those with multiple organ metastasis. Our results showed that surgery didn't benefit the patients with multiple metastasis. Thus, surgery is recommended for patients with distant single-organ metastasis, especially for lung metastasis, but not recommended for patients with distant multiple metastasis.

Surgical treatment has been a treatment of choice for early-stage NSCLC, and anatomical lobectomy has become the standard surgery for early NSCLC (20, 21). To date, no study has been conducted to investigate the role of lobectomy in the treatment of advanced lung cancer. Generally, stage IV lung cancer is treated with less invasive sublobectomy, and thus the patients can recover faster and receive subsequent systematic treatment, improving the prognosis. In our study, lobectomy significantly improved LCSS as compared to sublobectomy and pneumonectomy. The MST was 11 months in the sublobectomy group, 26 months in the lobectomy group, and 13 months in the pneumonectomy group. The 5-year survival rate in the sublobectomy, lobectomy, and pneumonectomy groups was 12.74, 28.01, and 15.99%, respectively. Wang et al. reported that the 5-year survival rate of NSCLC patients with limited metastasis was 21.1% after the surgery group, while it was 7.6% after radiotherapy and/or chemotherapy (13). In addition, their results showed that tumor pathology and surgical procedure had no significant influence on survival (13). Also, their results showed the surgery procedure did not affect survival, which might be ascribed to the small sample size. Our results showed the 5-year survival rate in the surgery group was slightly lower than that reported by Wang et al. (18.87 vs. 21.1%), but the 5-year survival rate in the lobectomy group was significantly higher (28.01 vs. 21.1%). In respect of site of metastasis, the prognosis of lobectomy patients was significantly better than that of sublobectomy or pneumonectomy patients among those with distant single-organ metastasis (lung, brain, bone, and liver), but the surgical procedure had no influence on the survival rate in patients with multiple metastasis. Our result suggests that lobectomy may be better than sublobectomy or pneumonectomy for patients with distant single-organ metastasis, but palliative treatment was recommended for patients with multiple metastasis, such as chemotherapy, radiotherapy, and targeted therapy.

## Limitations

This study also had several limitations. The protocols of chemotherapy, the dose of radiation, and information about targeted therapy were not recorded in the SEER program, which might also influence the prognosis. Although the data were collected from patients with a large sample size, the proportion of surgically treated patients was small (2.83%). Thus, the potential selection bias could not be totally excluded. More prospective controlled studies are thus needed in which the local and systemic therapies are integrated with the management of NSCLC with limited metastasis.

## Conclusions

In this study, SEER analysis revealed that NSCLC patients with single-organ metastasis rather than multiple metastases may benefit from local surgery. And, lobectomy is a better option than sublobectomy or pneumonectomy. In sum, lobectomy can significantly prolong the survival of NSCLC patients with single-organ metastasis, especially improve the LCSS and OS of NSCLC patients with lung oligometastatic.

## Data Availability Statement

The original contributions presented in the study are included in the article/supplementary materials, further inquiries can be directed to the corresponding author/s.

## Author Contributions

All authors listed have made a substantial, direct and intellectual contribution to the work, and approved it for publication.

## Conflict of Interest

The authors declare that the research was conducted in the absence of any commercial or financial relationships that could be construed as a potential conflict of interest.

## Abbreviations

NSCLC: Non-small cell lung cancer
SEER: Surveillance Epidemiology and End Results
LCSS: lung cancer-specific survival
OS: overall survival
pmNSCLC: primary metastatic non-small cell lung cancer
MST: median survival time.

## References

[1] Jemal A Bray F Center MM Ferlay J Ward E Forman D. Global cancer statistics. CA Cancer J Clin. (2011) 61:69–90. 10.3322/caac.20107 21296855

[2] Torre LA Bray F Siegel RL Ferlay J Lortet-Tieulent J Jemal A. Global cancer statistics, 2012. CA Cancer J Clin. (2015) 65:87–108. 10.3322/caac.21262 25651787

[3] Planchard D Popat S Kerr K Novello S Smit EF Faivre-Finn C. Metastatic non-small cell lung cancer: ESMO Clinical Practice Guidelines for diagnosis, treatment and follow-up. Ann Oncol. (2018) 29(Suppl. 4):iv192–237. 10.1093/annonc/mdy275 30285222

[4] Petrovich Z Yu C Giannotta SL O'Day S Apuzzo ML. Survival and pattern of failure in brain metastasis treated with stereotactic gamma knife radiosurgery. J Neurosurg. (2002) 97(5 Suppl.):499–506. 10.3171/jns.2002.97.supplement_5.0499 12507085

[5] Ramalingam S Belani C. Systemic chemotherapy for advanced non-small cell lung cancer: recent advances and future directions. Oncologist. (2008) 13(Suppl. 1):5–13. 10.1634/theoncologist.13-S1-5 18263769

[6] Rafii A Deval B Geay JF Chopin N Paoletti X Paraiso D. Treatment of FIGO stage IV ovarian carcinoma: results of primary surgery or interval surgery after neoadjuvant chemotherapy: a retrospective study. Int J Gynecol Cancer. (2007) 17:777–83. 10.1111/j.1525-1438.2007.00905.x 17367318

[7] Lin SZ Tong HF You T Yu YJ Wu WJ Chen C. Palliative gastrectomy and chemotherapy for stage IV gastric cancer. J Cancer Res Clin Oncol. (2008) 134:187–92. 10.1007/s00432-007-0268-z 17611776 PMC12161674

[8] Cheufou DH Welter S Chalvatzoulis E Christof D Theegarten D Stamatis G. Surgery of primary lung cancer with oligometastatic m1b synchronous single brain metastasis: analysis of 37 cases. Thorac Cardiovasc Surg. (2014) 62:612–5. 10.1055/s-0034-1377060 25136943

[9] Bella MJ Kowalewski J Dancewicz M Bławat P Szczesny TJ Chrzastek A. Results of surgical treatment of primary lung cancer with synchronous brain metastases. Kardiochir Torakochirurgia Pol. (2015) 12:14–7. 10.5114/kitp.2015.50562 26336472 PMC4520501

[10] Hanagiri T Takenaka M Oka S Shigematsu Y Nagata Y Shimokawa H. Results of a surgical resection for patients with stage IV non-small-cell lung cancer. Clin Lung Cancer. (2012) 13:220–4. 10.1016/j.cllc.2011.05.006 22138036

[11] Endo C Hasumi T Matsumura Y Sato N Deguchi H Oizumi H. A prospective study of surgical procedures for patients with oligometastatic non-small cell lung cancer. Ann Thorac Surg. (2014) 98:258–64. 10.1016/j.athoracsur.2014.01.052 24746441

[12] Casiraghi M Bertolaccini L Sedda G Petrella F Galetta D Guarize J. Lung cancer surgery in oligometastatic patients: outcome and survival. Eur J Cardiothorac Surg. (2020) 57:1173–80. 10.1093/ejcts/ezaa005 32091083

[13] Wang Z Gao SG Xue Q Guo XT Wang LX Yu X. Surgery of primary non-small cell lung cancer with oligometastasis: analysis of 172 cases. J Thorac Dis. (2018) 10:6540–6. 10.21037/jtd.2018.11.125 30746198 PMC6344694

[14] Wang Y Xie S He B. Effect of EGFR gene polymorphism on efficacy of chemotherapy combined with targeted therapy for non-small cell lung cancer in Chinese patients. Am J Cancer Res. (2019) 9:619–27. 30949415 PMC6448055

[15] Vaughn C Mychaskiw G 2nd Sewell P. Massive hemorrhage during radiofrequency ablation of a pulmonary neoplasm. Anesth Analg. (2002) 94:1149–51. 10.1097/00000539-200205000-00016 11973177

[16] Andratschke N Kraft J Nieder C Tay R Califano R Soffietti R. Optimal management of brain metastases in oncogenic-driven non-small cell lung cancer (NSCLC). Lung Cancer. (2019) 129:63–71. 10.1016/j.lungcan.2018.12.009 30797493

[17] Reck M Rabe KF. Precision diagnosis and treatment for advanced non-small-cell lung cancer. N Engl J Med. (2017) 377:849–61. 10.1056/NEJMra1703413 28854088

[18] Eberhardt WE Mitchell A Crowley J Kondo H Kim YT Turrisi A 3rd. The IASLC lung cancer staging project: proposals for the revision of the M descriptors in the forthcoming eighth edition of the TNM classification of lung cancer. J Thorac Oncol. (2015) 10:1515–22. 10.1097/JTO.0000000000000673 26536193

[19] Luketich JD Burt ME. Does resection of adrenal metastases from non-small cell lung cancer improve survival? Ann Thorac Surg. (1996) 62:1614–6. 10.1016/S0003-4975(96)00611-X 8957360

[20] Veluswamy RR Ezer N Mhango G Goodman E Bonomi M Neugut AI. Limited resection versus lobectomy for older patients with early-stage lung cancer: impact of histology. J Clin Oncol. (2015) 33:3447–53. 10.1200/JCO.2014.60.6624 26240229 PMC4606062

[21] Darling GE Allen MS Decker PA Ballman K Malthaner RA Inculet RI. Randomized trial of mediastinal lymph node sampling versus complete lymphadenectomy during pulmonary resection in the patient with N0 or N1 (less than hilar) non-small cell carcinoma: results of the American College of Surgery Oncology Group Z0030 Trial. J Thorac Cardiovasc Surg. (2011) 141:662–70. 10.1016/j.jtcvs.2010.11.008 21335122 PMC5082844

